# Phenotypic Diversity and Productivity of *Medicago sativa* Subspecies from Drought-Prone Environments in Mediterranean Type Climates

**DOI:** 10.3390/plants10050862

**Published:** 2021-04-24

**Authors:** Luis Inostroza, Soledad Espinoza, Viviana Barahona, Macarena Gerding, Alan Humphries, Alejandro del Pozo, Carlos Ovalle

**Affiliations:** 1 CRI-Quilamapu, Instituto de Investigaciones Agropecuaria, Chillán 3780000, Chile; 2 CRI-Raihuen Instituto de Investigaciones Agropecuaria, Cauquenes 3690000, Chile; soledad.espinoza@inia.cl (S.E.); viviana.barahona@inia.cl (V.B.); 3 Facultad de Agronomía, Universidad de Concepción, Chillán 3780000, Chile; mgerding@udec.cl; 4 South Australian Research and Development Institute (SARDI), Adelaide, SA 5000, Australia; alan.humphries@sa.gov.au; 5 Centro de Mejoramiento Genético y Fenómica Vegetal, Facultad de Ciencias Agrarias, Universidad de Talca, Talca 3460000, Chile; adelpozo@utalca.cl; 6 CRI-La Cruz, Instituto de Investigaciones Agropecuaria, La Cruz 228000, Chile

**Keywords:** canopy traits, forage yield, morphological traits, *Medicago sativa* subspecies, NDVI, SLA

## Abstract

The phenotypic diversity and productivity of a diverse alfalfa (*M. sativa* subspp.) panel of cultivars, landraces and wild relatives with putative drought tolerance were evaluated in two Mediterranean environments (central Chile and Southern Australia). In Chile, 70 accessions were evaluated in rainfed conditions and in Australia 30 accessions under rainfed and irrigated conditions, during three growing seasons. Large phenotypic variation was observed among and within subspecies for NDVI, stem length, intercepted PAR and forage yield. Principal component analysis indicated that the first two principal components (PC) accounted for 84.2% of total variance; fall dormancy, taxa, and breeding status were closely related to the agronomical performance of alfalfa accessions. Forage yield varied largely among accessions across years and locations. A linear relationship was found between annual forage yield and annual water added to the experiments (R^2^ = 0.60, *p* < 0.001). The GxE analysis for forage yield allowed the detection of the highest yielding accessions for each of the two mega-environments identified. The accessions CTA002 and CTA003 showed greater forage yield in both Chile and Australia environments. It is concluded that new breeding lines derived from crosses between cultivated alfalfa (*M. sativa* subsp. *sativa*) and wild relatives belonging to the primary (*M. sativa* subsp. *falcata*) and tertiary (*M. arborea*) gene pool, achieve outstanding agronomical performance in drought-prone environments.

## 1. Introduction

Mediterranean drought-prone environments are one the most threatened by climate change [1,2]. The increase in temperature along with the decline and larger interannual variability of rainfall, [3,4] are affecting dryland farming systems and their profitability, due to lower water availability for forage yield and therefore livestock production [5].

Alfalfa (*Medicago sativa*) is a perennial legume of high protein content and yield potential in Mediterranean type climates [6,7]. Its deep root system allows extending the growing season into early summer and autumn period and consequently increasing forage yield in rainfed environments [8].

There is evidence that the genetic diversity of *Medicago sativa* has been reduced during the domestication process; indeed, cultivated alfalfa has lost about 30% of its genetic diversity compared to wild populations [9]. It has been proposed that landraces and wild relatives of alfalfa encompass greater genotypic and phenotypic diversity than commercial cultivars, as well as adaptive traits to survive in extreme drought stress conditions [10,11,12,13].

Alfalfa belongs to the *Medicago sativa-falcata* complex, which includes subspecies that are partially sympatric and potentially freely hybridizing in nature [11]. The taxa encompassed in the complex contain both diploids and tetraploids, and gene flow can occur within and between ploidy levels. The most up-to-date list of taxa in the *M. sativa-falcata* complex include: *M. sativa* subsp. *caerulea* (2n), *M. sativa* subsp. *falcata* (2n and 4n), *M. sativa* subsp. x *hemicycla* (2n), *M. sativa* subsp. *glutinosa* (2n), *M. sativa* subsp. *sativa* (4n), *M. sativa* subsp. x *varia* (4n), and *M. sativa* subsp. *glomerata* (4n) [14]. These subspecies represent the primary gene pool for alfalfa improvement [10,11,14]. Recently, Humphries et al. [10] pointed out that primary gene pool offers great potential for alfalfa drought tolerance improvement. Indeed, most of the subspecies are originally from arid zones or drought-prone environments of Eurasia. For instance, *M. sativa* subsp. *caerulea* germplasm collected from the Caspian shore is reported to be late flowering, high yielding, adapted to arid conditions, and over-grazing. *M. sativa* subsp. *falcata* hybridizes readily with wild populations of alfalfa (*M. sativa* subsp. *sativa*) at the tetraploid level. These two taxa have hybridized extensively, giving rise to stabilized introgressant populations, which have been assigned to the collective taxon *M. sativa* subsp. x *varia*. Many (by no means all) populations of subsp. *falcata* and *varia* occur in arid areas, and are potential sources of drought-resistant germplasm [10]. Some wild *M. sativa* subsp. *falcata* populations have shown lower stomatal conductance [15] and higher leaf chlorophyll content under severe drought compared to domesticated alfalfa [16], indicating ability to delay leaf senescence and retain photosynthesis.

*Medicago arborea* L. is a leguminous shrub well-adapted to the Mediterranean area. It has many traits of potential use in alfalfa breeding; can reach up to 4 m tall; is remarkably drought-resistant and is the longest-lived *Medicago* species. In addition, *M. arborea* also has large seeds, disease resistance and morphological traits that could restructure alfalfa [17]. Recently, researchers have attempted the introgression of favorable traits from *M. arborea* genome to *M. sativa* subsp. *sativa* such as drought tolerance [10,17,18]. Hybrids between *M. sativa* subsp. *sativa* and *M. arborea*, which have been given the common name Alborea, have been produced in the USA and Australia [17,18]. A study by Tani et al. [18] showed that at the seedling stage, Alborea plants exhibited higher stem elongation rate, photosynthetic rate, stomatal conductance, and transpiration rate than their parent *M. sativa* subsp. *sativa* and *M. arborea* accessions.

The genetic diversity of crop wild relatives conserved in world’s germplasm banks has not been sufficiently used for crop improvement [19]. According to Dempewolf et al. [20], the use of crop wild relatives in cultivar development have been limited due to: (i) the existence of sufficient variation in the cultivated germplasm of some crops; (ii) differences in ploidy and other hybridization barriers between wild species and cultivated germplasm; (iii) inferiority of wild relatives of crops for desired traits; (iv) insufficient phenotypic and genotypic data on wild relative accessions; (v) inadequate human or financial resources to carry out the necessary research and development.

In this article we studied the phenotypic diversity and productivity of a diverse alfalfa panel composed by cultivars, landraces and wild relatives, all of them with putative drought tolerance [10], in two Mediterranean environments (central Chile and Southern Australia). The objectives were (a) to compare morpho-physiological traits of four *M. sativa* subspecies (*M. sativa* subsp. *sativa*, *M. sativa* subsp. *caerulea*, *M. sativa* subsp. *varia* and *M. alborea*); (b) to evaluate the broad sense heritability (*H*^2^) of traits; (c) to evaluate their productivity in dryland Mediterranean conditions. The ultimate goal is to select candidate materials to be incorporated into a selection program for drought tolerant varieties for the Mediterranean zone of Chile and Australia. The accessions and lines described in this paper are available from the Australian Pastures Genebank, (https://apg.pir.sa.gov.au/gringlobal/search.aspx, accessed on 21 April 2021) and further information on this project is available at www.projectwebsite, accessed on 21 April 2021.

## 2. Results

### 2.1. Plant Survival, Canopy Traits and Forage Yield

The alfalfa diverse panel included four *M. sativa* subspecies (*M. sativa* subsp. *sativa*, *M. sativa* hybr. (Alborea), *M. sativa* subsp. *caerulea*, and *M. sativa* subsp. *varia*; hereafter sativa, alborea, caerulea, and varia, respectively; Supplementary Table S1). Within the sativa pool, 20 out of 51 phenotypically characterized accessions were cultivars originated in China, Kazakhstan, Australia, and the USA. Two cultivars belonging to varia were also included in the diversity panel. The remaining accessions were landraces (36) and pre-bred lines (11) developed by crossing cultivars of sativa with alfalfa wild relatives (Supplementary Table S1).

The phenotypic characterization was performed over three years in South Australia (2016–2019) and Chile (2017–2020). The first six months was considered the establishment year and therefore was not included in the analysis. In terms of growing seasons, data from the first and second growing season after the establishment year were considered in both South Australia and Chile. Plant survival was similar among the four subspecies, but there was important variability within subspecies (Table 1). Phenotypic variation was observed among and within subspecies for normalized difference vegetation index (NDVI), plant height, fraction of intercepted photosynthetic active radiation (FIPAR) and annual forage yield; *alborea* showed the highest NDVI, SL, FIPAR and forage yield values (Table 1). In all phenotypic traits evaluated, excepting plant survival, more than 50% of the total phenotypic variance was attributed to the alfalfa accessions component (Table 1); broad sense heritability ranged between 0.53 and 0.85. Plant survival was highly affected by the environmental condition; it showed a H^2^ value of 0.10.

**Figure 1:**
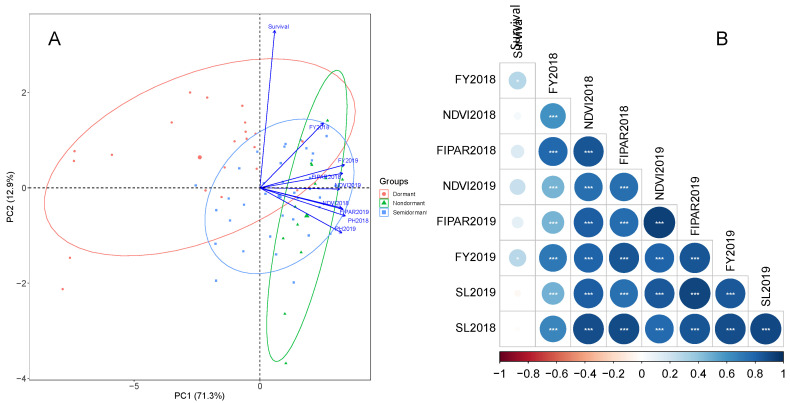
Biplot (**A**) and correlogram (**B**) of the first two principal components (PC1 and PC2) for the principal component analysis of nine traits evaluated in the alfalfa diversity panel in Chile. The traits are forage yield (FY), in the first (FY2018) and second (FY2019) growing seasons, plant survival (Survival), Normalized Difference Vegetation Index (NDVI), fraction of intercepted photosynthetic active radiation (FIPAR), and plant height (PH) evaluated in 2018 and 2019 growing seasons. In the correlogram * and *** refer to *p* < 0.05 and *p* < 0.001, respectively.

Principal component analysis indicated that the first two principal components (PC) accounted for 84.2% of total variance; PC1 and PC2 explained 71.3 and 12.9% of the variability, respectively (Figure 1). Each variable, except forage yield in the first growing season (2018–2019) and plant survival, contributed 13% in average to PC1. Whereas the traits with greater contribution to PC2 were plant survival (74.1%) and forage yield during the first growing season (12.6%). The forage yield in the first and second growing seasons were significantly correlated with all phenotypic traits evaluated (*p* < 0.05) with r-values ranging between 0.27 (plant survival) and 0.85 (FIPAR) (Figure 1B).

Fall dormancy, taxon, and breeding status determined the agronomic performance of alfalfa accessions. To observe the fall dormancy effects, the alfalfa accessions were assigned to three categories of fall dormancy: dormant type (fall dormancy 0–3), semidormant type (fall dormancy 4–7), and nondormant type (fall dormancy 8–10). The dormant type showed the lowest PC1 values and the highest PC2 (Figure 1A), which were associated with lower and higher values of forage yield and plant survival, respectively (Figure 1A). The nondormant type exhibited the highest PC1 values and the lowest PC2. In both categories, there were some outstanding alfalfa accessions. For instance, accessions APG45675 and CTA008 (dormant type), reached middle-high values of PC1. Accessions CTA003, APG45669 and APG 58574 (nondormant type) reached middle-high values of PC2, which is associated with high annual forage yield and high plant survival. Semidormant accessions exhibited PC1 and PC2 values that were between those of dormant and nondormant types.

As mentioned before, PC1 mostly described agronomic performance and PC2 plant survival. The caerulea accessions showed the lowest agronomic performance in comparison to other subspecies. Sativa accessions were distributed across the entire PC1, which reflects a high level of genetic diversity for the evaluated traits. The varia and alborea accessions reached PC1 values within the sativa pool (Supplementary Figure S2A). In regard to breeding status, some landraces (APG6567, APG45669, APG40234, AltaSierra12) and pre-bred lines (CTA003, CTA004, CTA008) exhibited similar and even better agronomic performance than some cultivars (Supplementary Figure S2B).

### 2.2. Morphological Traits

Alfalfa subspecies exhibited similar morphological expression, excluding caerulea accessions, which showed the lowest values of StemDM, LeavesDM, LSratio, SLA, Leaf Size, StemD and StemL (Table 2). The sativa pool showed the broadest range of variation in all morphological traits evaluated. Excluding caerulea, alborea accessions showed between 10 and 15% higher values of StemDM, LeavesDM, LSratio, SLA, Leaf Size, StemD and StemL than the other subspecies (Table 2). Excluding StemD and StemL, morphological traits exhibited low genetic control; broad sense heritability varied between 0.10 (LeavesDM) and 0.47 (StemDM).

**Figure 2:**
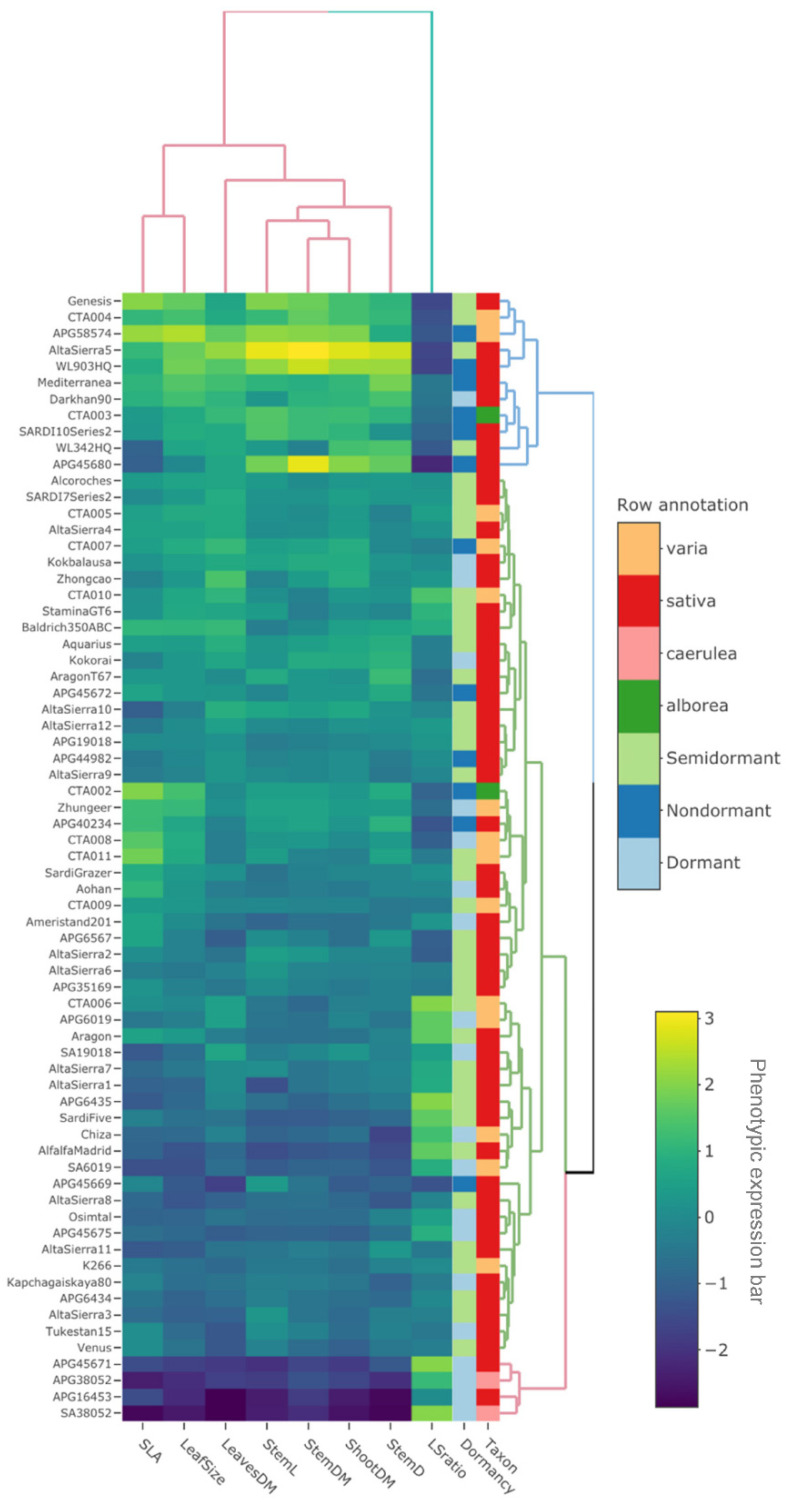
Heatmap visualization of two-way cluster analyses for eight morphological traits (column tree) and 70 alfalfa accessions (row tree) evaluated in the second growing season (2019–2020) in Mediterranean drought-prone environment of Chile. Row annotation indicates taxon and fall dormancy. The greater values in phenotypic expression bar indicate greater phenotypic values in a given trait. Morphological traits: shoot dry matter (ShootDM), stem dry matter (StemDM), leaves dry matter (LeavesDM), LeavesDM to StemDM ratio (LSratio), specific leaf area (SLA), stem length (StemL), stem diameter (StemD), and leaf size (LeafSize).

**Figure 3:**
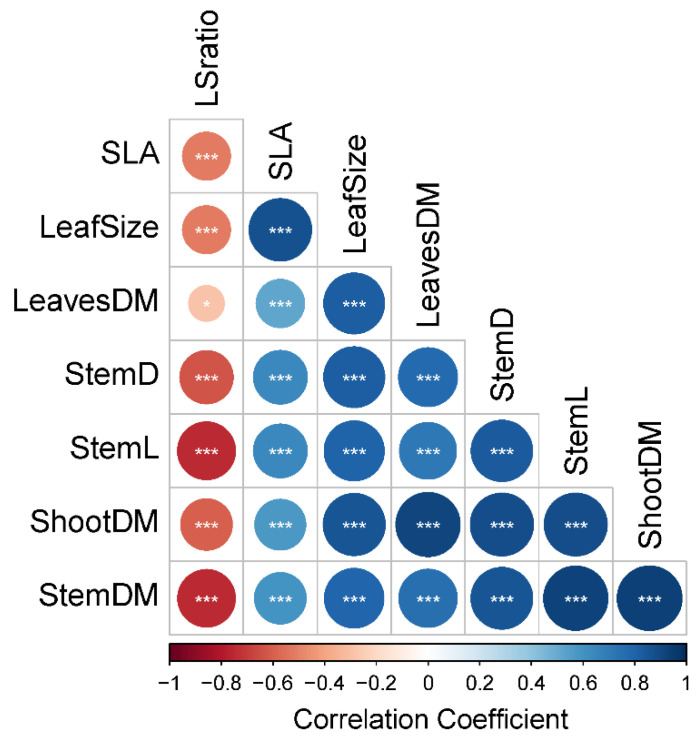
Correlogram of eight morphological traits evaluated in 70 alfalfa accessions in the second growing season (2019–2020) in a Mediterranean drought-prone environment of Chile. In the correlogram * and *** refer to *p* < 0.05 and *p* < 0.001, respectively. Morphological traits: shoot dry matter (ShootDM), stem dry matter (StemDM), leaves dry matter (LeavesDM), LeavesDM to StemDM ratio (LSratio), specific leaf area (SLA), stem length (StemL), stem diameter (StemD), and leaf size (LeafSize).

Two-way cluster analysis (heatmap) identified two groups for morphological traits. One of them included only LSratio. All other morphological traits were clustered together. Three clusters were found for alfalfa accessions (Figure 2). At accession level, Cluster I, included caerulea accessions and two sativa landraces (APG45671 and APG16453). This cluster was characterized for showing the lowest values of LeavesDM, StemDM, StemL, StemD, Leaf Size and SLA, but high values of LSratio. On the other hand, Cluster III was characterized for including three subspecies (sativa, varia and alborea) and accessions with different breeding status. Cluster III included cultivars (WL903HQ, Sardi10, Darkhan90, and Genesis), pre-bred lines (CTA004 and CTA003) and landraces (APG45680, AltaSierra5, Mediterranea, and APG58574). Alfalfa accessions included in Cluster III were characterized for exhibiting morphological characteristics totally opposite to Cluster I (Figure 2). Morphological traits were all significantly correlated among them. The LSratio was the unique morphological trait negatively correlated with each of the other traits (Figure 3).

### 2.3. Forage Yield in Drought-Prone Environments in Chile and Australia

**Figure 4:**
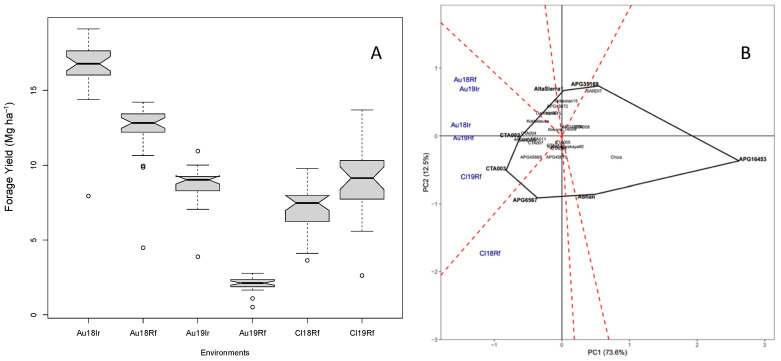
Frequency distribution (box plot; **A**) and GGE biplot (**B**) of annual forage yield evaluated in 30 alfalfa accessions in drought-prone environments of Chile and Australia during the first and second growing season after the establishment year. GGE biplot corresponds to the first two principal components (PC1 and PC2) for the principal component analysis of annual forage yield evaluated in six environments: rainfed experiment in Australia in 2017–2018 (Au18Rf) and 2018–2019 (Au19Rf) growing seasons, irrigated experiment in Australia in 2017–2018 (Au18Ir) and 2018–2019 (Au19Ir) growing seasons, and rainfed experiment in Chile in 2018–2019 and 2019–2020 growing seasons (Cl18Rf and Cl19Rf).

Annual forage yield data corresponds to the first and second growing seasons after the establishment year, in both Australia and Chile. In Australia, during the first growing season (2017–2018), forage yield under irrigated condition was close to 35% higher than rainfed condition. However, during the second growing season (2018–2019), the forage yield in irrigated condition was almost three times higher than that observed in rainfed condition. In Chile, the average forage yield was 7.2 and 8.9 Mg ha^−1^ during the first and second growing season, respectively (Figure 4A). In all environments, except in Au18Rf, the forage yield showed broad range of variation among alfalfa accessions (Figure 4A). The GGE analysis accounted for 86.1% for the total variance in forage yield and the GGE biplot analyzed the Accession x Environment (AxE) interaction (Figure 4B). Environments were clustered in two polygons or mega-environments (ME). In ME including Au18Rf, Au19Ir, Au18Ir, Au19Rf, and Cl19Rf pre-bred lines CTA002 and CTA003 showed greater forage yield. In the ME including Cl18Rf, the landrace APG6567 expressed the highest forage yield.

**Figure 5:**
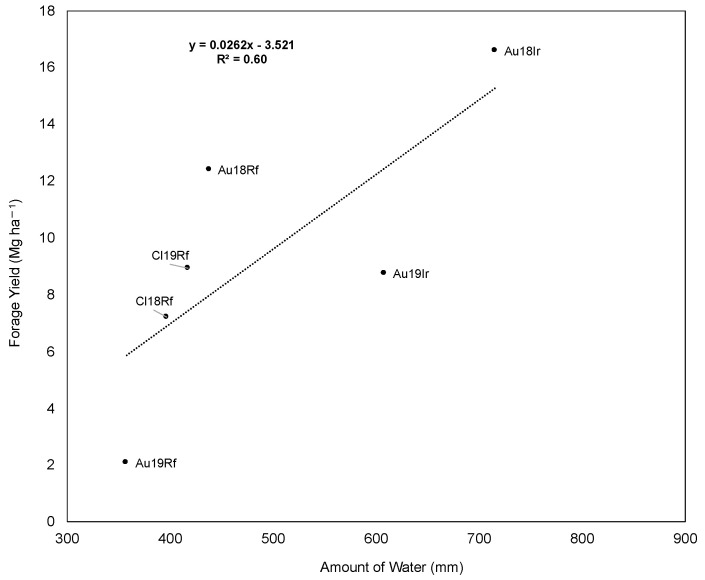
Relationship between annual forage yield evaluated in Mediterranean environments of Chile and Australia and amount of water (rainfall or rainfall + irrigation) added to the experimental plots. Environments were rainfed experiment in Australia in 2018 (Au18Rf) and 2019 (Au19Rf) growing seasons, irrigated experiment in Australia in 2018 (Au18Ir) and 2019 (Au19Ir) growing seasons, and rainfed experiment in Chile in 2018 and 2019 growing season (Cl18Rf and Cl19Rf).

The total amount of water added to each experimental site in every growing season was estimated (rainfall + irrigation). A linear relationship was found between annual forage yield and annual water added to the experiments (R^2^ = 0.60, *p* < 0.001; Figure 5).

## 3. Discussion

This work confirms the existence of a broad phenotypic diversity among accessions of the *M. sativa-falcata* complex [11,12,14,21,22,23,24], but it also adds new evidence of the genetic contribution of alfalfa wild relatives for improving agronomic performance of cultivated alfalfa in Mediterranean drought-prone environments. Furthermore, new insights about the outstanding genetic contribution of *Medicago arborea* to the *M. sativa-falcata* complex were observed at field condition in pre-bred lines.

Fall dormancy is a critical trait for successful cultivation of alfalfa across the world [25,26] since it contributes to alfalfa adaptation/survival in harsh winter environments. In this work, nondormant accessions exhibited higher forage yield than dormant (Figure 1A), which is coincident with results reported in other works [25,26]. In environments with mild temperatures during autumn–winter, nondormant alfalfa genotypes produce more herbage in autumn, resume shoot growth earlier in spring, and initiate shoot regrowth quickly after harvest in summer [27]. As expected, the dormant accessions showed higher plant survival than the nondormant accessions. However, there were some nondormant (CTA003) and semidormant (APG6567) accessions with similar plant survival to dormant alfalfa accessions (Figure 1A). It is important to highlight the dormant accessions APG45675 and CTA008, which expressed similar agronomic performance to nondormant alfalfa (Figure 1A). Accession APG45675 is a sativa landrace grown by farmers in the Bolivian Altiplano. Whereas, accession CTA008 is a varia accession bred from the cross between a falcata wild material originally from Kazakhstan and the Australian cultivar Sardi-Grazer [10]. These two dormant accessions could be used for breeding high-yielding alfalfa cultivars in cold environments [26].

### 3.1. Phenotypic Diversity

A large body of literature supports the abundant genetic diversity within the *M. sativa-falcata* complex [10,11,13,14,22,23,24,26,28]. However, genetic diversity used for breeding has been mostly based on cultivated populations. The use of wild populations in breeding programs has been fairly limited [29]. In this work, accessions CTA008 and CTA004, which are varia pre bred-lines, represent the great contribution of the primary gene pool on sativa genome, because it exhibited one of the highest plant survivals with suitable agronomic performance in Mediterranean drought-prone environments (Supplementary Figure S2B).

In the last decades, great efforts have been undertaken in order to produce hybrids between *M. sativa* and *M. arborea*, known as the *alborea* hybrids, which are expected to perform better in drought-prone environments [18]. In this agronomic study, the pre-bred line CTA003, which corresponds to an *alborea* hybrid derived from the cross between *M. sativa* subsp. *sativa* cv. Genesis and *M. arborea* [10], showed one of the highest DM yields and plant survival (Figure 1). Additionally, the accession CTA003 exhibited similar DM yield to cv. Genesis but with higher plant survival in Mediterranean drought-prone environment. This insight could be considered as empirical evidence of the drought tolerance improvement transferred from *M. arborea* to *M. sativa*. Tani et al. [18], showed that at seedling stage, alborea hybrids exhibited higher stem elongation rate, photosynthetic rate, stomatal conductance, and transpiration rate (E) than sativa and arborea accessions under drought stress, indicating that the hybrids correspond to a drought tolerance ideotype [30,31,32,33,34,35].

Morphological characterization revealed that leaf and stem traits are highly correlated within the *M. sativa-falcata* complex (Figure 2 and Figure 3). Additionally, ShootDM (an estimator of forage yield) forage yield was significantly associated with SLA, leaf size and StemD, which coincide with results of other works [36]. From an agronomic point of view, the alfalfa LSratio has been used as a positive estimator of forage quality, owing to the greater quality of leaves relative to stems [32,37,38]. The LSratio performed as a differentiator trait of the agronomic categories identified by the cluster analysis. For instance, Cluster I, which grouped low-yielding accessions (Figure 2) and small-sized plants (lowest values of LeavesDM, StemDM, ShootDM, LeafSize, StemD and StemL), showed the highest values of LSratio. In contrast, Cluster III, which grouped high-yielding materials and large-sized plants, showed lower values of LSratio. Clusters I and III showed LSratio values of 0.99 and 2.0 on average, respectively. In others works, high forage quality alfalfa cultivars or populations had shown LSratio near to one [37,38,39]. Thus, pre-bred lines (CTA004 and CTA003) and landraces (APG58574, APG45680, Mediterranea, and Altasierra5) grouped in Cluster III could putatively be considered as high forage quality materials.

The relationship between LSratio and forage yield has been scarcely studied. In this work, the LSratio was negatively correlated with all measured morphological traits including ShootDM (Figure 3). Based on this association, a negative trade-off between forage yield and LSratio could be expected. Recently, Annicchiarico [40] pointed out that selection for high-yielding alfalfa populations in Italy did not have any impact on LSratio. However, the range of variation in LSratio of those materials was between 0.89 and 1.0.

Alfalfa cultivars grown in optimal conditions have shown SLA values ranging between 250 and 460 cm^2^ g^−1^ [41], which is in agreement with the range of variation observed in this work, excluding the values of the diploid materials (caerulea; Table 2). SLA is a morphological trait strongly associated with alfalfa drought tolerance [36,42]. Erice et al. [36] reported genotypic differences in SLA expression; drought tolerant accessions reduced their SLA under drought conditions, whereas the sensitive accession did not modify it. Mickky et al. [42] also observed the reduction in SLA under drought condition, which was associated with increased leaf thickness and reduced phloem and xylem area. All these modifications could be considered as water saving physiological mechanisms. In this work, accessions belonging to *varia* and *alborea* exhibited higher values of SLA, which would support the putative drought tolerance associated with these two subspecies [10,14,17,18]. Accessions with higher values of SLA (344 cm^2^ g^−1^, in average) were landraces APG58574 (varia), APG40234 (sativa), and Altasierra5 (sativa); pre-bred lines CTA011, CTA008, CTA004 (varia), and CTA002 (alborea); cultivars Zhungeer (varia) and Genesis (sativa).

### 3.2. Forage Yield and Accession by Environment Interaction (AxE)

Forage yield in Mediterranean drought-prone environments varied broadly among accessions and across years and locations (Figure 4A). In Australia, irrigation had a large impact on annual forage production, particularly in the third growing season (Figure 4A and Figure 5), where annual forage yield was three-fold higher in irrigated relative to rainfed condition. This could be explained by the rainfall conditions during the 2019 growing season, since South Australia recorded the driest year in more than a decade, with 170 mm less than the long-term average (528.3 mm). Similar response to irrigation was reported by Li and Su [43] in alfalfa grown under a gradient of water treatments in Northern China. Additionally, forage yield throughout locations and years was significantly related to the amount of rainfall or water added to the experiments (Figure 5), which is the typical agronomic responses of crops grown in Mediterranean environments [8,44,45]. The GGE analysis accounted for AxE interaction. Two mega-environments were identified (Figure 4B). However, one of them included all environments, except Cl18Rf. This information is relevant because the Chilean and Australian environments are largely different in their edaphic condition. Therefore, our results suggest that climate has a greater weight on phenotypic expression than soil. This supports the idea that cultivars developed with specific adaptability to Mediterranean environments of Chile or Australia, will perform well in other Mediterranean regions of the world. Recently, Annicchiarico [40] demonstrated the importance of AxE on alfalfa cultivars development. He pointed out that genetic gains on forage yield are significantly higher when cultivars are developed with specific adaptability to target environments. Our results agree with this statement, since the accessions characterized in this work were selected based on their history of drought tolerance or specific adaptability to drought-prone environments [10]. For instance, the landrace APG6567 (*varia*), was top yielding in Chile because it was collected in arid zones of Spain [10]. In the same way, the alborea hybrids (CTA002 and CTA003), which exhibited the highest forage yield in Australia (2018 and 2019 growing seasons) and Chile (2019 growing season).

From a plant breeding point of view, the inheritance of morphological and agronomic traits evaluated in this alfalfa diverse panel was similar to that reported in other works. For instance, forage yield heritability values around 0.5 has been also reported by Julier et al. [46] and Ray et al. [47]. The LSratio heritability values were similar to values reported by Guines et al. [48]. However, we found a broad range of plant height heritability values in literature; values of 0.09, 0.29, and 0.45 have been reported [46,47,48]. Differences in heritability among different studies may well arise from differences in the population type and size and/or differences in experiment error size. Furthermore, all heritability values used to compare our results were estimated in alfalfa breeding populations.

In conclusion, the wild and pre-bred alfalfa germplasm studied in this work increased the morphological diversity in the *M. sativa-falcata* complex and allowed to find candidate materials to be incorporated into a selection program for drought tolerant varieties for the Mediterranean zone of Chile and Australia. Additionally, this work revealed that new breeding lines derived from crosses between cultivated alfalfa and wild relatives belonging to the primary (*M. sativa-falcata* complex) and tertiary (*M. arborea*) gene pool achieve outstanding agronomic performance in drought-prone environments.

## 4. Materials and Methods

### 4.1. Plant Material

A set of 70 alfalfa accessions with putative drought and salinity tolerance was identified using curator knowledge and acquired through the CWR-alfalfa research project supported by Crop Trust (https://www.cwrdiversity.org/partnership/alfalfa-pre-breeding-project-2/, accessed on 21 April 2021). The set included landraces, cultivars and advanced genetic lines (here after accessions) originated from Kazakhstan, Azerbaijan, Spain, Australia, USA, and Chile (Table S1; [10]).

### 4.2. Experimental Sites and Plants Establishment

In Chile, seeds of 70 accessions (Supplementary Table S1) were sown in 200-holes germination trays containing peat moss as substrate (Kekkila, Finland). Substrate was daily irrigated and periodically fertilized with a solution of 1.1 g L^−1^ Phostrogen (Bayer, Cambridge, UK). Seedlings were inoculated with a suspension of *Sinorhizobium meliloti* (strain WSM2141) and grown for two months under greenhouse condition. One week before transplanting, seedlings were moved to a shelter for hardening. On August 2017, seedlings were transplanted at the Cauquenes Research Station of INIA-Chile (35°57′ S; 72°19′ W). Seedlings were arranged in plots of five rows of 2.5 m length separated by 40 cm among them (125 plants per plot). Before transplanting, the soil was prepared using a chisel plow and disc harrows. Plots received 200 kg ha^−1^ of triple phosphate (45% P_2_O_5_), 2000 kg ha^−1^ of CaCO_3_, 100 kg ha^−1^ of potassium sulphate (50% K_2_O and 54% SO_4_) and 20 kg ha^−1^ of boronatrocalcite (11% B). The experiment was arranged in an α-lattice design with three replicates. Each replicate had five incomplete blocks including 14 accessions. The soil is classified as Ultic Palexeralfs, with a pH of 5.7 (1:2.5 in water, 0–20 cm), organic matter content of 2.7% and available N, P, and K content at the top 20 cm of 772 18, 17, and 250 mg kg^−1^, respectively. The experiment was managed under rainfed condition with 5 or 6 months of negative water balance (Evapotranspiration (ET) > rainfall) during a growing season (Supplementary Figure S1).

In Australia, 30 accessions (Supplementary Table S1) were sown on 26 August 2016 in 1.2 × 2.8 m plots (3.36 m^2^) at sowing rate of 7 kg ha^−1^. Seeds were inoculated with Group AL rhizobium prior to sowing. The experiment was established with two blocks at the Waite Research Institute of University of Adelaide (34°58′ S, 138°38′ E). The blocks differed in their water regime, with one managed under rainfed conditions whilst the other block was irrigated. Similar to Chile, the rainfed experiment included 5 or 6 months with negative water balance (Evapotranspiration (ET) > rainfall) during a growing season (Supplementary Figure S1).

Plots were arranged in an augmented (unreplicated) row and column design. The experiments included 12 rows with eight accessions each (columns). The sativa cultivar SARDI 7 Series 2 was grown once in every eight accessions as a highly repeated check to remove spatial variation. The soil was fine sandy loam of the non-sodic Urrbrae series. Soil pH (in CaCl_2_) was 6.0 with negligible calcium carbonate [49].

The irrigated experiment had subsurface drip irrigation, with two lines running 50 cm apart, 20 cm beneath each plot, and drip intervals of 50 cm. Irrigation was applied monthly at 30 mm between November and May, increasing to twice per week when temperature was above 35 °C.

### 4.3. Phenotypic Characterization

In Chile, several morphological, physiological and agronomic traits were evaluated, which are described in the following sections. In Australia, only forage yield and plant height were determined.

### 4.4. Plant Survival and Forage Yield

Plant survival was evaluated by counting the number of surviving plants per plot at the beginning of the fourth growing season (spring 2020) and expressing the result as a percentage of the establishment density (125 plants per plot).

Forage yield was evaluated by cutting 1 m^2^ of each plot at 5 cm aboveground. Samples were oven-dried with forced air ventilation at 70 °C until reaching constant weight for dry matter determination. Two and three biomass cuts were performed during 2018 and 2019 growing seasons, respectively. The dates of cuts were 13 September 2018, 21 November 2018, 3 September 2019, 24 October 2019, and 19 December 2019. Annual forage yield was determined adding the cuts per years. Plant height (PH) was evaluated in every plot one day before forage yield determination with a 1-m ruler.

### 4.5. Morphological Traits

Five randomly selected stems per plot were collected. Stem length was measured with a ruler and stem diameter was determined in the middle section of the stem with a digital caliper. Five fully expanded trifoliated leaves were selected for leaf area (LA), petiole length (PL) and dry weight of leaves determination. LA and PL were measured with leaf area meter device (MK2, Delta-T Devices, Cambridge, UK) and digital caliper, respectively. Then, leaves were collected in paper bags for drying. Specific leaf area (SLA) was determined as the relationship between LA and leaves dry weight. Finally, leaves (LeavesDM), stem (StemDM), and shoot dry matter (ShootDM = StemDM + LeavesDM), were determined. Additionally, the LeavesDM:StemDM ratio was calculated. Dry matter contents of leaves and stems were determined after drying of samples at 70 °C in forced air ventilation oven until they reached a constant weight. Morphological characterization was performed at the beginning of the fourth growing season, when plants expressed their higher vigor (28 October 2020).

### 4.6. Canopy Characterization

The fraction of intercepted photosynthetic active radiation (FIPAR) was evaluated in each plot with a ceptometer, which includes a one meter long probe with 64 PAR sensors and a BF5 reference PAR sensor (SunScan canopy analyzer); three determinations at the bottom of each plot were taken, placing the ceptometer in parallel to the crop row. FIPAR measurements were taken one day before the forage yield determination between 11:00 and 16:00 h, in 2018 and 2019 growing seasons. The NDVI was determined simultaneously with FIPAR measurements, with a handheld spectroradiometer (GreenSeeker, Trimble, Sunnyvale, CA, USA). Measurements were performed on clear days before the sampling for forage yield, passing the sensor 60 cm above the top of the canopy. The NDVI corresponds to the differences between the reflectance (R) in the near-infrared (760 nm) and red (660 nm) band, and is calculated as: (R760 − R660)/(R760 + R660).

### 4.7. Statistical Analysis

A phenotypic linear mixed model including correlated error for accounting fine-scale spatial variation among experimental units was implemented in the Chilean and Australian data sets. Both experiments were arranged in rows and columns for modeling spatial variation. The model was implemented to estimate the variance components and the best linear unbiased prediction (BLUP) using the restricted maximum likelihood method within the ASReml-R package [50] in R software (https://www.r-project.org/, accessed on 21 April 2021) using the following equation:

(1) $$Y_{ik}=\mu +r_{k}+g_{i}+\varepsilon_{ik}$$

where *Y_ik_* is the phenotypic value of *i*th alfalfa accession (*g*) in the *k*th replicate (*r*), *μ* is the overall population mean, *r* is the fixed effect of replicate, *g* is the random effect of the alfalfa accession ~IDD(0,*σ*_*g*^2), and ε the random experimental error ~IDD(0,*σ*_*ε*^2). Year effect and its interactions with other terms were not considered in the model. The first-order autoregressive anisotropic covariance structure (AR1 x AR1) was used to model spatial variation in row and column directions. This model is reportedly adequate to account for spatial variation in yield trials of cultivars and breeding lines [51,52]. For the unreplicated Australian experiments, the standard check information (Sardi 7) was used for calculating variance components and BLUPs in accordance with Piepho and Williams [53]. In brief, the structure of the model is similar to the model described above, but the alfalfa accession effect (*g*) was simultaneously modeled as fixed and random effects when standard check and other accessions were adjusted, respectively.

The variance components of the Chilean data sets were used to estimate the broad-sense heritability (*H*^2^) on a plot-mean basis, which was calculated as follows:

(2) $$H^{2}=\frac{\sigma_{g}^{2}}{\sigma_{g}^{2}+\frac{\sigma_{\varepsilon }^{2}}{r}}$$

Predicted means were used to perform multivariate analyses. The relationship among phenotypic traits and alfalfa accessions and the effect of fall dormancy, taxon, and status of breeding on agronomic performance were studied with principal component analysis (PCA) using the packages FactoMinR [54] and factoextra [55] in R software. The morphological diversity expressed by the *Medicago* subspecies was explored with a two-way clustering analysis using heatmaply package in R software [56].

The annual forage yields of alfalfa accessions evaluated in Chile and Australia were used to explore the accession by environment interaction (AxE). Annual forage yield was the total throughout a growing season. In Chile and Australia, the growing season was extended from spring (September) to autumn (March). Two growing seasons were considered in this analysis, the first and second after the establishment year. Full combinations of locations and growing seasons were considered as single environment. Therefore, the analysis was performed with six environments: rainfed experiment in Australia in 2017–2018 (Au18Rf) and 2018–2019 (Au19Rf) growing seasons, irrigated experiment in Australia in 2017–2018 (Au18Ir) and 2018–2019 (Au19Ir) growing seasons, and rainfed experiment in Chile in 2018–2019 and 2019–2020 growing seasons (Cl18Rf and Cl19Rf). The AxE interaction was explored with the GGE biplot method using the GGEbiplot package in R software [57,58].

## Figures and Tables

**Table 1:** Mean ± standard deviation and broad sense heritability (*H*^2^ ± SE) of plant survival, normalized difference vegetation index (NDVI), plant height, fraction of intercepted photosynthetic active radiation (FIPAR) and dry matter production, evaluated in an alfalfa diversity panel including accessions belonging to four subspecies *M. sativa* subsp. *sativa*, *M. sativa* subsp. *caerulea*, *M. sativa* subsp. *varia* and *M. sativa* subsp. *alborea*. Phenotypic characterization was performed during two growing seasons (2018–2019 and 2019–2020) in a Mediterranean drought-prone environment in Chile.

Taxon	NDVI		Plant Height (cm)		FIPAR		Forage Yield (Mg ha^−1^)		Plant Survival (%)
	2018/19	2019/20	2018/19	2019/20	2018/19	2019/20	2018/19	2019/20	
*M.s. hybr. (alborea)* (* n = 2)	0.62 ± 0.11	0.80 ± 0.07	52.9 ± 0.1	43.3 ± 6.8	0.88 ± 0.01	0.87 ± 0.04	8.88 ± 0.1	11.65 ± 2.8	84.2 ± 12.8
*M.s. caerulea* (n = 2)	0.35 ± 0.05	0.32 ± 0.01	9.3 ± 4.2	8.3 ± 5.4	0.035 ± 0.01	0.13 ± 0.09	4.10 ± 0.04	1.25 ± 0.2	79.3 ± 9.2
*M.s. sativa* (n = 50)	0.59 ± 0.12	0.79 ± 0.08	39.8 ± 10.8	36.3 ± 10.3	0.69 ± 0.21	0.79 ± 0.18	6.67 ± 1.8	8.83 ± 2.6	85.2 ± 5.9
*M.s. varia* (n = 14)	0.56 ± 0.10	0.76 ± 0.10	37.6 ± 9.5	34.0 ± 10.3	0.68 ± 0.17	0.72 ± 0.23	7.15 ± 0.9	8.75 ± 2.2	87.4 ± 4.5
*H* ^2^	0.85 ± 0.02		0.67 ± 0.08		0.65 ± 0.07		0.53 ± 0.09		0.10 ± 0.01

* n is the number of accessions in each taxon.

**Table 2:** Mean ± standard deviation and broad sense heritability (*H*^2^ ± SE) of eight morphological traits evaluated in an alfalfa diversity panel including accessions belonging to four subspecies *M. sativa* subsp. *sativa*, *M. sativa* subsp. *caerulea*, *M. sativa* subsp. *varia* and *M. sativa* subsp. *alborea*. Phenotypic characterization was performed during the second growing season (2019–2020) in Mediterranean drought-prone environment of Chile.

Taxon	ShootDM (g)	StemDM (g)	LeavesDM (g)	LSratio (g g^−1^)	SLA (g cm^−2^)	StemL (cm)	StemD (mm)	LeafSize (cm^2^)
*M.s. hybr. (alborea)* (* n = 2)	3.70 ± 0.57	1.78 ± 0.25	1.93 ± 0.32	1.12 ± 0.06	319.7 ± 70.8	50.4 ± 5.7	3.05 ± 0.10	96.07 ± 9.8
*M.s. caerulea* (n = 2)	1.23 ± 0.60	0.45 ± 0.28	0.78± 0.32	2.31 ± 0.81	86.3 ± 16.9	25.1 ± 3.2	1.69 ± 0.21	10.81 ± 6.8
*M.s. sativa* (n = 50)	3.06 ± 0.86	1.35 ± 0.51	1.69 ± 0.39	1.40 ± 0.43	242.5 ± 50.5	42.3 ± 7.9	2.71 ± 0.39	67.62 ± 22.8
*M.s. varia* (n = 14)	3.18 ± 0.71	1.36 ± 0.47	1.82 ± 0.30	1.58 ± 0.58	269.4 ± 64.9	43.1 ± 7.0	2.63 ± 0.31	79.10 ± 25.4
*H* ^2^	0.28 ± 0.10	0.47 ± 0.12	0.10 ± 0.01	0.30 ± 0.01	0.23 ± 0.07	0.62 ± 0.07	0.52 ± 0.06	0.26 ± 0.02

Morphological traits: shoot dry matter (ShootDM), stem dry matter (StemDM), leaves dry matter (LeavesDM), LeavesDM to StemDM ratio (LSratio), specific leaf area (SLA), stem length (StemL), stem diameter (StemD), and leaf size (LeafSize).

## Data Availability

We don’t have a link. However, if somebody needs our data set, they can contact to corresponding authors.

## Supplementary Materials

The following are available online at https://www.mdpi.com/article/10.3390/plants10050862/s1, Table S1: Passport information of the alfalfa diversity panel and sites where accessions were evaluated. Figure S1: Water balance in Chile (A) and Australia (B) of growing seasons in which the alfalfa diverse panel was cultivated. Reference evapotranspiration (ET) was calculated according to FAO standards; monthly accumulated rainfall (Rainfall). Meteorological data in Chile and Australia were obtained from Agromet-INIA (https://agrometeorologia.cl, accessed on 21 April 2021) and the Australian Bureau of Meteorology (http://www.bom.gov.au/?ref=hdr, accessed on 21 April 2021), respectively. Figure S2: Biplot of the first two principal components (PC1 and PC2) for the principal component analysis of nine traits evaluated in the alfalfa diversity panel in Chile. The traits are forage yield (FY), in first (FY2018) and second (FY2019) growing seasons, plant survival (Survival), Normalized Difference Vegetation Index (NDVI), fraction of intercepted photosynthetic active radiation (FIPAR), and plant height (PH) evaluated in 2018 and 2019 growing seasons. Biplots are highlighting the effect of taxon (A) and status of breeding (B).
