# Supplementary material for: Phenotypic Diversity and Productivity of Medicago sativa Subspecies from Drought-Prone Environments in Mediterranean Type Climates
Source: Plants (Basel). 2021 Apr 24;10(5):862. doi: 10.3390/plants10050862 (PMC8146503; doi:10.3390/plants10050862)
Supplement: Supplementary file 1 [file plants-10-00862-s001.zip › plants-1177023-SI.pdf]

plants

MDPI

Supplementary file:

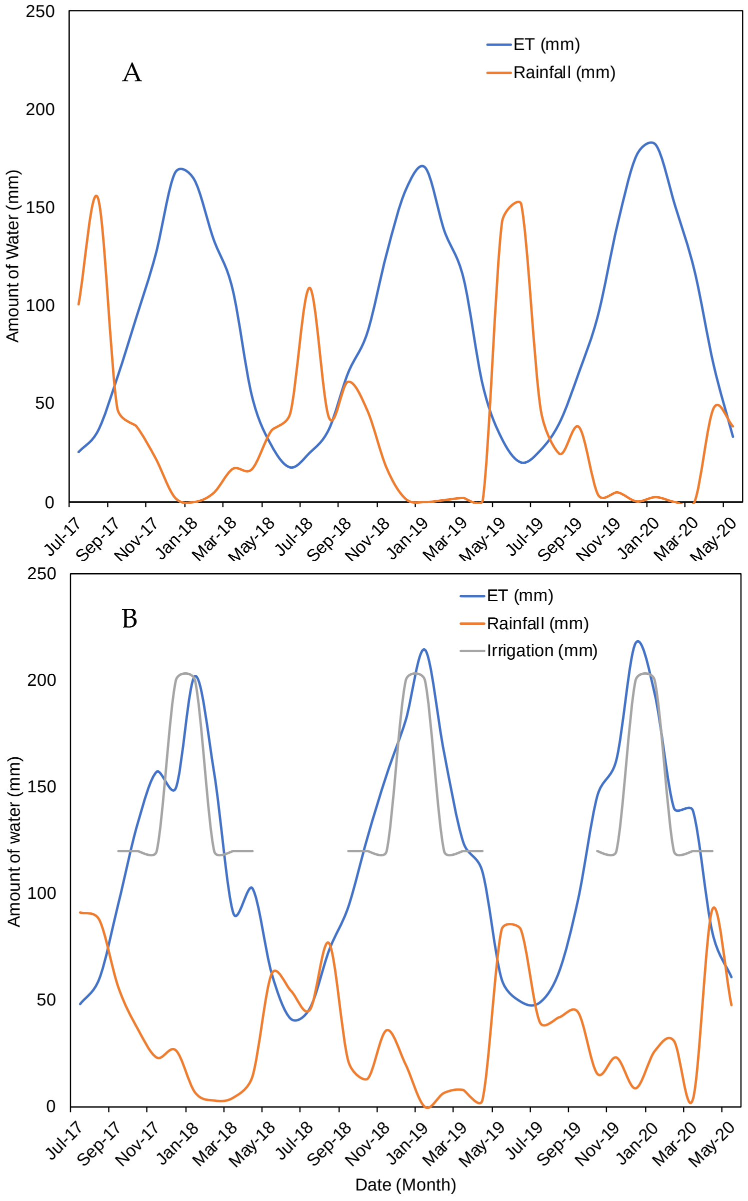

Figure S1. Water balance in Chile (A) and Australia (B) of growing seasons in which the alfalfa diverse panel was cultivated. Reference evapotranspiration (ET) was calculated according to FAO standards; monthly accumulated rainfall (Rainfall). Meteorological data in Chile and Australia were obtained from Agromet-INIA (https://agrometeorologia.cl) and the Australian Bureau of Meteorology (http://www.bom.gov.au/?ref=hdr), respectively.

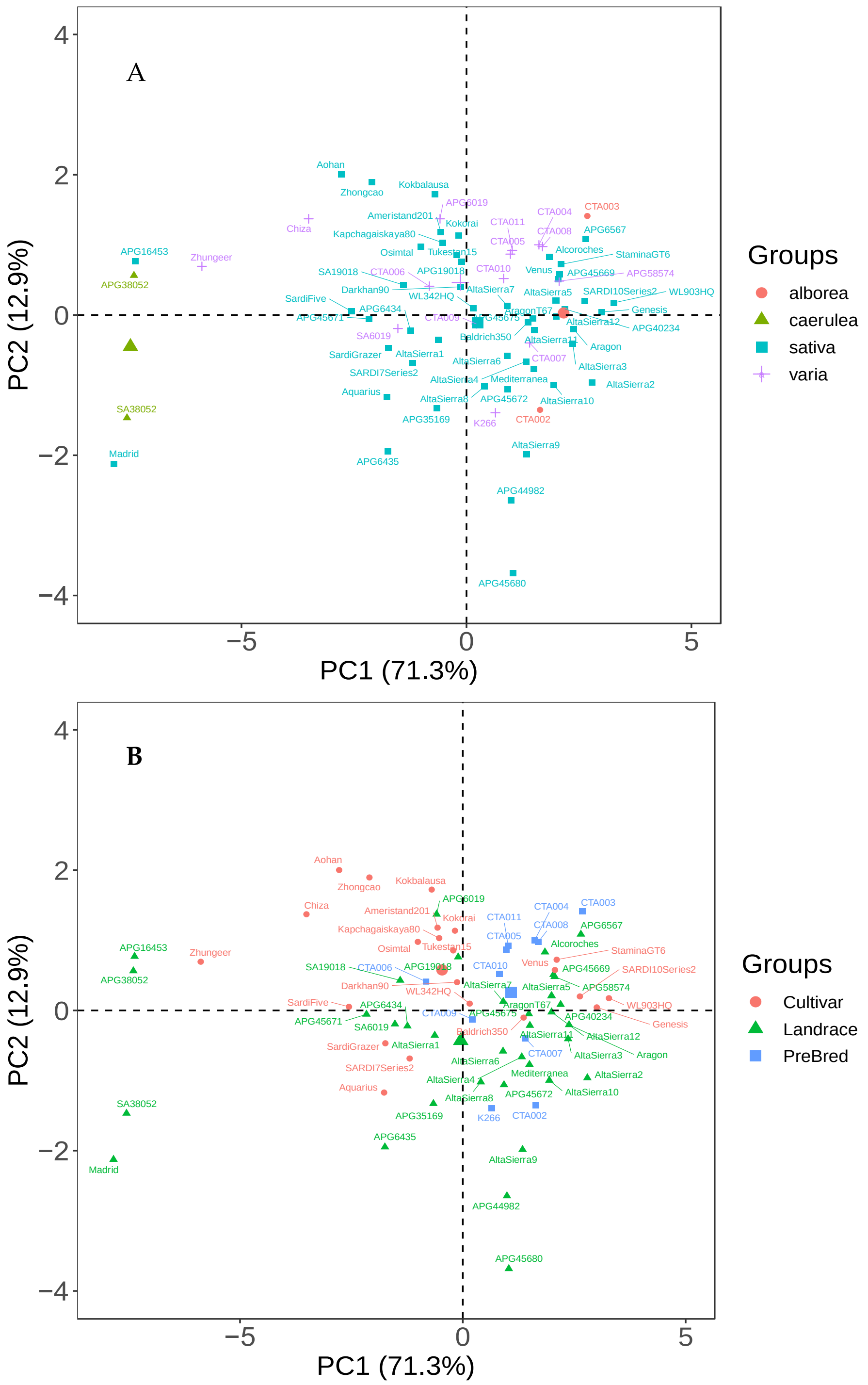

Figure S2. Biplot of the first two principal components (PC1 and PC2) for the principal component analysis of nine traits evaluated in the alfalfa diversity panel in Chile. The traits are: Forage yield (FY), in first (FY2018) and second (FY2019) growing seasons, plant survival (Survival), Normalized Difference Vegetation Index (NDVI), fraction of intercepted photosynthetic active radiation (FIPAR), and plant height (PH) evaluated in 2018 and 2019 growing seasons. Biplots are highlighting the effect of Taxon **(A),** and status of breeding **(B).**
